# Efficacy and safety of adjuvant radiation therapy in localized adrenocortical carcinoma

**DOI:** 10.3389/fendo.2023.1308231

**Published:** 2024-01-08

**Authors:** Luming Wu, Jiayi Chen, Tingwei Su, Lei Jiang, Yimin Han, Cui Zhang, Weiwei Zhou, Yiran Jiang, Xu Zhong, Weiqing Wang

**Affiliations:** ^1^ Department of Endocrine and Metabolic Diseases, Shanghai Institute of Endocrine and Metabolic Diseases, Ruijin Hospital, Shanghai Jiao Tong University School of Medicine, Shanghai, China; ^2^ Department of Radiation Oncology, Ruijin Hospital, Shanghai Jiao Tong University School of Medicine, Shanghai, China

**Keywords:** adrenocortical carcinoma, radiation therapy, efficacy & safety, localized ACC, adjuvant (chemo)radiotherapy

## Abstract

**Context:**

Adrenocortical carcinoma (ACC) is rare and have high rates of recurrence and mortality. The role of adjuvant radiation therapy (RT) in localized ACC was controversial.

**Methods:**

We conducted a retrospective study in our center between 2015 and 2021 to evaluate the efficacy and safety of adjuvant RT in localized ACC. Overall survival (OS) and disease-free survival (DFS) were estimated using the Kaplan-Meier method. Cox proportional hazards regression models were used to estimate the independent risk factors. Adverse events associated with RT were documented according to the toxicity criteria of the radiation therapy oncology group (RTOG) and the common terminology criteria for adverse events (CTCAE v5.0).

**Results:**

Of 105 patients with localized ACC, 46 (43.8%) received adjuvant RT after surgery. The median radiation dose was 45.0Gy (range:30.0-50.4) and median follow up time was 36.5 (IQR: 19.7-51.8) months. In comparison to the no adjuvant RT group, patients with adjuvant RT had better 3-year OS (87.9% vs 79.5%, P=0.039), especially for patients with ENSAT I/II stage (P=0.004). Adjuvant RT also improved the median DFS time from 16.5months (95%CI, 12.0-20.9) to 34.6months (95%CI, 16.1-53.0). Toxicity of RT was generally mild and moderate with six grade 3 events.

**Conclusions:**

Postoperative adjuvant RT significantly improved OS and DFS compared with the use of surgery alone in resected ACC patients. Although this retrospective study on RT in localized ACC indicates that RT is effective in ACC, its findings need to be prospectively confirmed.

## Introduction

Adrenocortical carcinoma (ACC) is rare and highly malignant, with a poor prognosis (1). The therapeutic options are limited, complete surgical removal remains the only curative treatment option (2). However, the cumulative recurrence rate is high (30%-75%) (3–5), leading to a poor 5-year survival rate (6). Therefore, adjuvant therapy is needed to improve cllinical outcomes.

Adjuvant therapy, including mitotane, systemic chemotherapy, radiation therapy (RT), and immune therapy have been explored in various settings. Mitotane is still the only medication specifically approved by FDA for the treatment of ACC (7). Given the rarity of the disease, there is a paucity of prospective randomized studies to guide clinical practice in the use of these adjuvant therapies. Habra et al. (8) found adjuvant RT within 3 months of primary surgical resection did not improve clinical outcomes. While older anecdotal reports involving from 2 to 5 patients each have concluded radiotherapy is ineffective for ACC (9, 10). Therefore, only 10% to 14% of patients with ACC receiving radiation in United States. Hence, efficacy and safety of adjuvant RT in localized ACC remains unclear. Until now, the majority of recent studies have suggested that adjuvant radiation therapy can significantly reduce local recurrences (11–15). However, data are conflicting regarding overall recurrence-free and overall survival.

Therefore, the aim of the retrospective study was to investigate patients with ACC treated with adjuvant radiation therapy after gross total resection and to evaluate its efficacy and tolerance.

## Methods

### Patients

Patients who received adjuvant radiation therapy from 2015 to 2021 were retrospectively diagnosed as ACC in Ruijin Hospital, Shanghai Jiao Tong University School of Medicine. We selected patients with stage I to III or oligometastatic stage IV who underwent definitive surgical resection (including oligo-metastectomy for those with stage IV), all patients were staged according to the European Network for the Study of Adrenal Tumors staging system pre-operation. Patients with ENSAT IV or received RT after recurrence were excluded. Two patients were excluded due to unknown death time. After exclusion, 46 ACC patients were finally included in this study. All of these patients treated with adjuvant RT in three months from the date of surgery in our center. All patients were treated with intensity-modulated RT. Doses to the primary target ranged from 30.0 to 50.4 Gy (median, 45 Gy). Patients who received adjuvant RT were then matched to control subjects who received surgery with curative intent but without adjuvant RT from 2015 to 2021 in our center. The no adjuvant RT group initially consisted of 59 patients matched to the RT group patients on the basis of resection margin [no evidence of tumor in resection margins (R0), microscopic evidence of tumor in margins (R1), macroscopic residual disease (R2), or unknown status of margins (Rx)] and disease stage at diagnosis. This study was approved by the board of medical ethics of Ruijin Hospital, Shanghai Jiao Tong University School of Medicine. Written informed consents were obtained from all patients included in the study.

### Outcome assessment

A period of 3 months was selected as the mandatory follow-up time. Overall survival (OS) time was calculated from the date of first surgery to the date of death or last follow-up. Disease Free survival (DFS) time was calculated from the date of first surgery to the date of first recurrence (whether local or distant) or last follow-up. Patient data were censored at the last follow-up date if recurrence or death did not occur.

### Documentation of adverse events

Medical records of adverse events related to RT were reviewed. Retrospective scoring of all adverse events was performed based on the toxicity criteria of the radiation therapy oncology group (RTOG) and the common terminology criteria for adverse events (CTCAE v5.0) (16).

### Statistical analysis

Overall and Disease-free survival probabilities were estimated using the Kaplan-Meier product limit method. Compare the distribution between subjects and control subjects using logarithmic rank test statistics. The Cox proportional hazards regression model is used to estimate the risk ratio (HR) of time event endpoints. Statistical analysis was performed with SPSS 25.0 software (IBM, Armonk, NY, USA). For all statistical tests, P values <0.05 were considered statistically significant.

## Results

### Patient characteristics

A total of 105 patients with localized ACC were available to assess based on the inclusion and exclusion criteria. All patients underwent radical adrenalectomy with curative intent. Of these, 46 (43.8%) patients received postoperative adjuvant RT. Median age at initial diagnosis was 47 years (IQR: 34, 58 years) for the total cohort. Median tumor size was 8.5 cm (IQR: 6.0, 11.3 cm). And most patients were female (51.4%).

The baseline characteristics of patients were shown in **Table 1**. There was no significant difference in gender, age, tumor diameter, Ki67, ENSAT stage, cortisol production or receipt of mitotane. During the follow-up, 25/105 patients were known to have died and 79/105 patients had a recurrence. 5(10.9%) patients treated with adjuvant RT were died, compared to 20 (33.9%) patients without adjuvant RT (P=0.006). 23 (50.0%) patients were identified with recurrence in the adjuvant RT group, compared to 46 (78.0%) patients in the no adjuvant RT group (P=0.003). A period of 3 months was selected as the mandatory follow-up time. Follow-up time was not significantly different between two groups (39.2(19.1, 55.8) vs 34.1(21.2, 49.5), P=0.722).

**Table 1:** Baseline Characteristics of Patients.

	No adjuvant RT (n=59)	Adjuvant RT (n=46)	P value
Gender, Male/Female	31/28	20/26	0.357
Age	55.0 (44.8, 61.0)	42.0 (31.0, 55.5)	0.115
Primary tumor diameter, cm	8.9 (5.8, 12.6)	8.0 (6.5, 10.3)	0.746
ENSAT			
I	5	2	0.552
II	35	35	
III	8	8	
Unknown	11	1	
Surgical margins			
R0	47	35	0.748
R1	5	6	
Rx	7	5	
Ki67	20.0 (10.0, 40.0)	15.0 (10.0, 40.0)	0.627
Cortisol production, n	19	11	0.583
Mitotane, n	36	24	0.310
Chemotherapy, n	14	9	0.519
Immune therapy, n	4	3	0.906
Death, n	20	5	0.006
Recurrence, n	46	23	0.003
Follow-up, months	39.2 (19.1, 55.8)	34.1 (21.3, 49.6)	0.722

Data are presented as median (IQR).

### Survival outcomes

**Figure 1:**
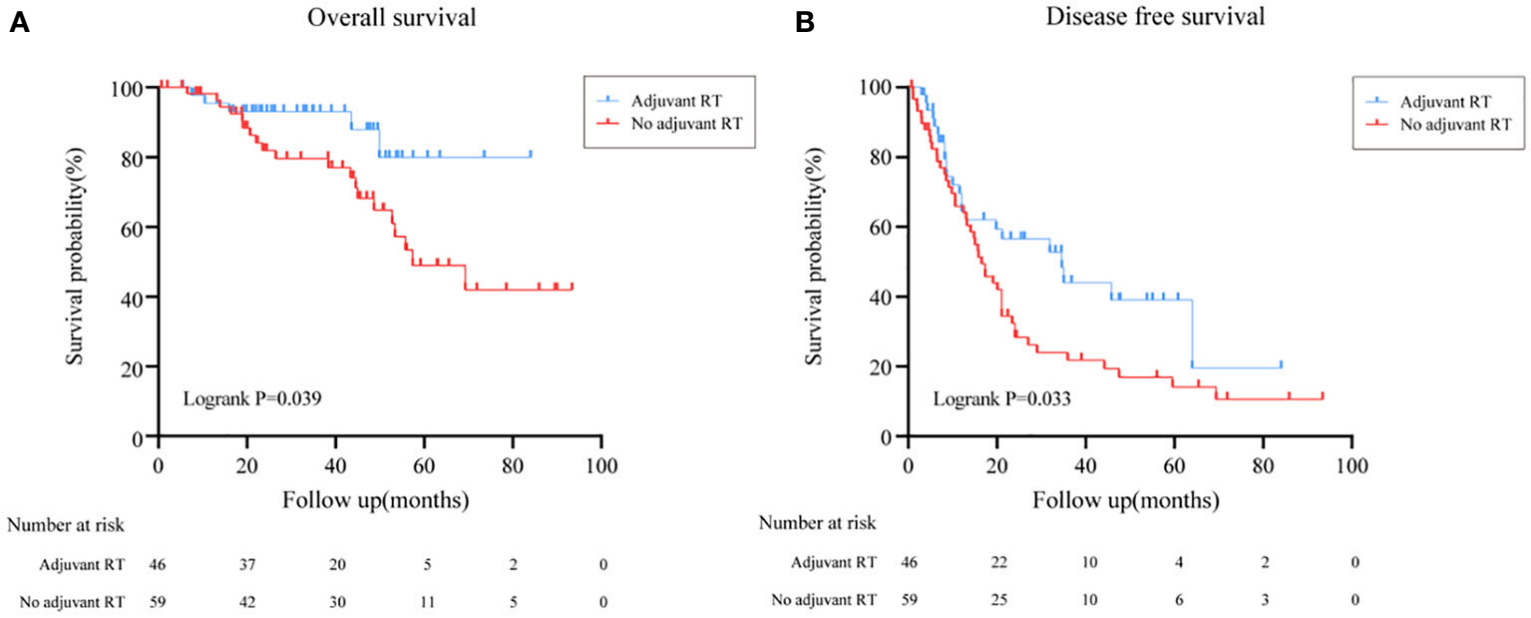
Overall survival and Disease free survival in all patients treated with adjuvant RT or with no adjuvant RT. **(A)** Overall survival in all patients treated with adjuvant RT or with no adjuvant RT. **(B)** Disease free survival in all patients treated with adjuvant RT or with no adjuvant RT.

The overall survival (OS) distributions between two groups were significantly different (Logrank P=0.039) (**Figure 1A**). Compared with 79.5% in the no adjuvant RT group, OS estimates at 3 years were 87.9% in the adjuvant RT group. In univariable analysis, stage III disease, cortisol production were positive associated with worse OS significantly, while adjuvant RT was associated with improved OS (HR, 0.368, 95%CI: (0.137, 0.986), P=0.047). After adjustment with ENSAT stage, adjuvant RT was still associated with improved survival outcomes significantly (HR, 0.293, 95%CI: (0.107, 0.798), P=0.016) (**Table 2**).

**Table 2:** Univariable and Multivariable Cox Regression Analysis for OS in Patients With ACC.

	Univariable		Multivariable	
	HR (95%CI)	P value	HR (95%CI)	P value
Age ≥55years	0.699 (0.291, 1.678)	0.423	–	–
Gender (Female)	1.302 (0.590, 2.874)	0.514	–	–
Tumor diameter ≥10cm	0.715 (0.276, 1.853)	0.490	–	–
Ki67				
<10%	Reference		Reference	
≥10%	2.962 (0.991, 8.857)	0.052	–	–
ENSAT stage				
I/II	Reference		Reference	
III	5.265 (2.066, 13.417)	0.001	6.131 (2.338, 16.075)	<0.001
Cortisol	5.392 (1.497, 19.422)	0.010	–	–
Adjuvant RT	0.368 (0.137, 0.986)	0.047	0.293 (0.107, 0.798)	0.016

HR, Hazard ratio; RT, Radiation therapy.

Variables with P<0.05 in univariable analysis were included in the multivariable Cox regression.

**Figure 2:**
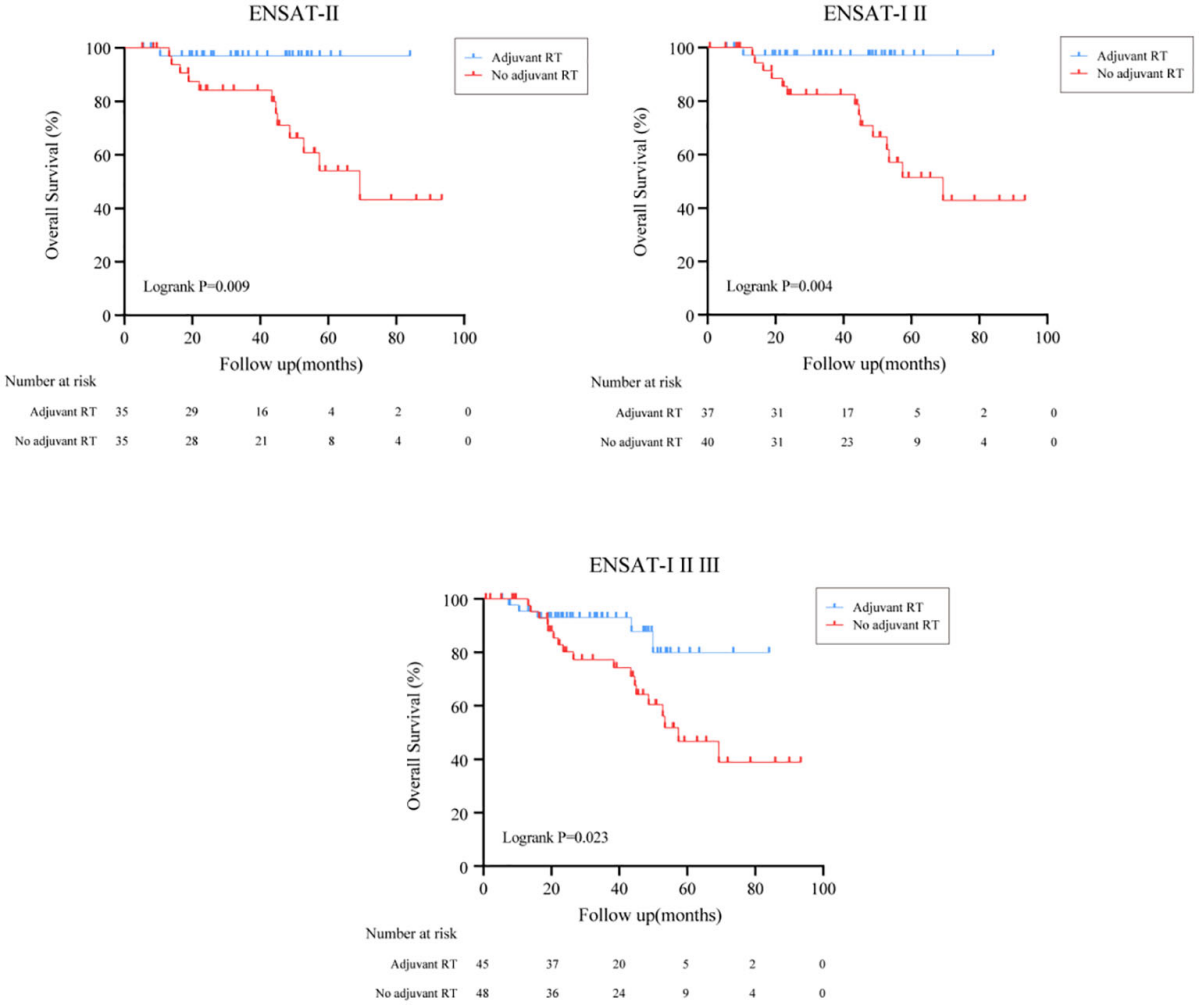
Effect of adjuvant RT on survival outcomes in patients with different ENSAT stage.

In subgroup analysis, the effect of adjuvant RT on survival outcomes according to ENSAT stage was then examined as shown in **Figure 2**. After surgical resection, we found that ACC patients who received adjuvant RT had better survival outcomes in definitive ENSAT II, ENSAT I/II and ENSAT I/II/III subgroup (Logrank P<0.05) (**Figure 2**).

### Recurrence outcomes

**Figure 3:**
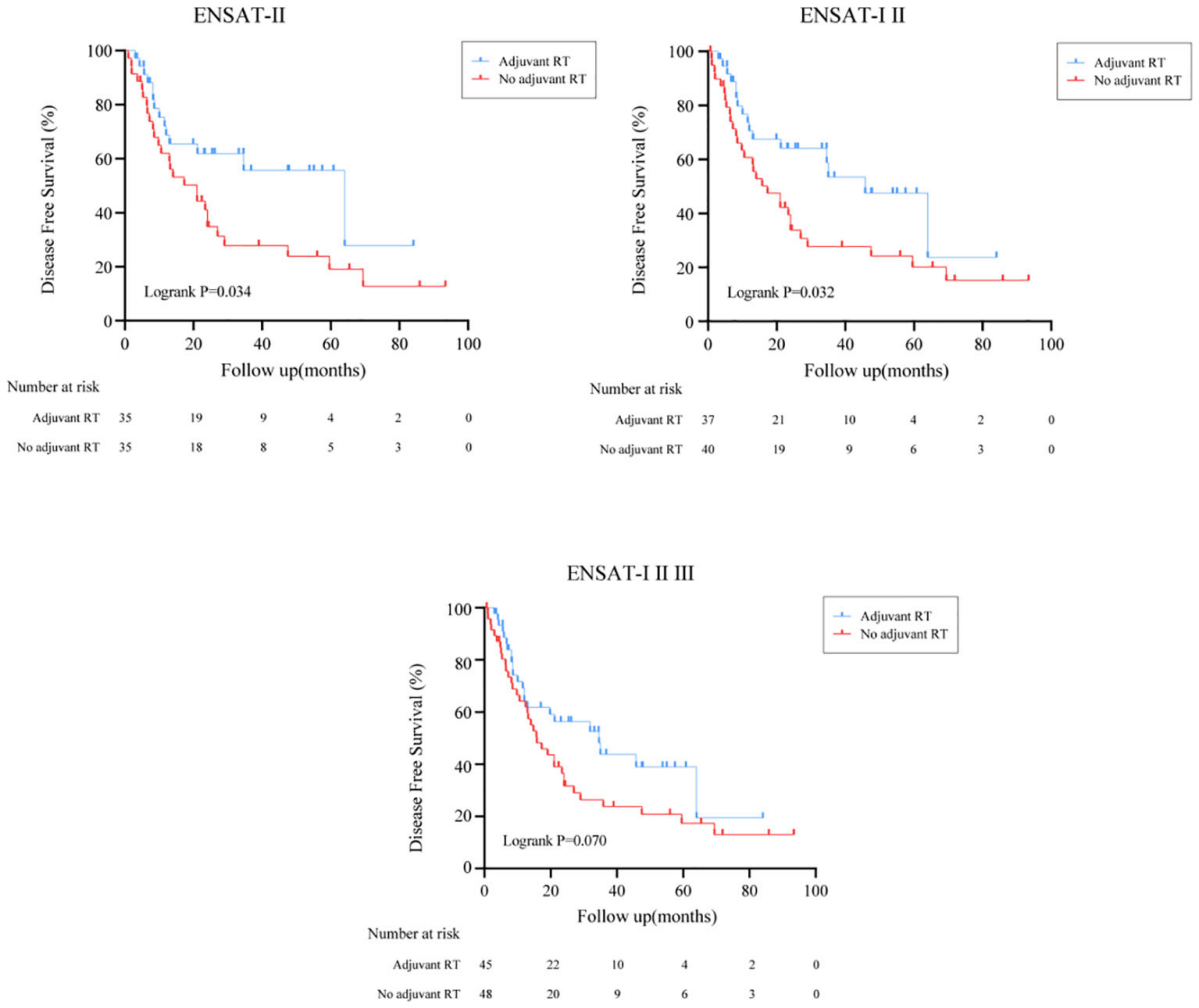
Effect of adjuvant RT on DFS in patients with different ENSAT stage.

The effect of adjuvant RT on recurrence outcomes was examined in all prespecified subgroups. Adjuvant RT improved the median of DFS time from 16.5 months (95%CI: (13.5, 19.5)) to 34.6 months (95%CI: (16.0, 53.1), Logrank P=0.033) (**Figure 1B**). Moreover, the recurrence outcomes of patients with ENSAT I/II was improved significantly (Logrank P=0.032) (**Figure 3**).

The results of the univariable and multivariable Cox regression model for DFS was shown on **Table 3** shows in patients with localized ACC who underwent complete surgical resection. In univariable analysis, Ki67(>10%), stage III disease and cortisol production were associated with decreased DFS (P<0.05). Adjuvant RT associated with increased DFS (P<0.05). Meanwhile, Age (>55years), female and tumor diameter (>10cm) were not significantly associated with DFS (all P>0.05). However, in the multivariable Cox analysis, after adjustment with Ki67 and ENSAT stage, adjuvant RT was independently associated with improved OS (P=0.016), while the association was absence in adjuvant RT and improved DFS (P=0.164).

**Table 3:** Univariable and Multivariable Cox Regression Analysis for DFS in Patients With ACC.

	Univariable		Multivariable	
	HR (95%CI)	P value	HR (95%CI)	P value
Age ≥55years	0.706 (0.417, 1.196)	0.196	–	–
Gender (Female)	1.024 (0.634, 1.652)	0.924	–	–
Tumor diameter ≥10cm	0.959 (0.538, 1.711)	0.888	–	–
Ki67				
<10%	Reference		Reference	
≥10%	1.952 (1.091, 3.491)	0.024	1.836 (0.977, 3.451)	0.059
ENSAT stage				
I/II	Reference		Reference	
III	2.180 (1.158, 4.106)	0.016	2.183 (1.108, 4.303)	0.024
Cortisol	3.147 (1.659, 5.968)	0.001	–	–
Radiation therapy	0.582 (0.352, 0.963)	0.035	0.666 (0.376, 1.180)	0.164

HR, Hazard ratio; RT, Radiation therapy.

Variables with P<0.05 in univariable analysis were included in the multivariable Cox regression.

### Toxicity

The documented adverse events associated with RT were mostly mild or moderate, according to the RTOG and CTCAE criteria. The most frequent toxicities were low grade intestinal adverse events (including nausea and vomiting) and leukopenia (**Table 4**). A single case of grade 3 intestinal adverse event with nausea occurred and five leukopenia grade 3 events were documented, respectively.

**Table 4:** Adverse events according to the RTOG and CTCAE criteria.

Adverse event	Grade 1	Grade 2	Grade 3
Intestinal	17	0	1
Leukopenia	13	4	5
Hepatic	2	1	0
Fatigue	1	0	0

## Discussion

Our retrospective study represents the cohort of patients with localized ACC treated with RT. This data indicate that RT is of benefit for patients underwent primary tumor resection followed with adjuvant RT. The toxicity reported was moderate and within the expected range. Our findings suggested that postoperative RT seem to be a valid adjuvant therapy option in localized ACC patients.

The role of adjuvant RT in ACC has been controversial. Early series exploring the potential benefit of adjuvant radiation drew conflicting conclusions (17–20). Study of the German ACC registry in 2006 had only 14 patients in the RT group (21), the results suggested an improvement in local control with adjuvant RT. However, a subsequent retrospective analysis in 2013, with 16 patients in the adjuvant RT group and 32 in the non-RT group, did not show any benefit result in local control and overall survival (8). The retrospective study in University of Michigan showed overall survival was not significantly different between two groups with the use of case-matched cohort analysis (22). However, Gharzai et al. found that adjuvant RT after gross resection of ACC improved the local RFS, all RFS and overall survival in a retrospective propensity-matched analysis (23). Compared with this study, a total of 105 patients with localized ACC were available in our study, and 46 (43.8%) patients received postoperative adjuvant RT. In comparison to the no adjuvant RT group, patients with adjuvant RT had better 3-year OS and DFS, especially for patients with ENSAT I/II stage.

High-risk ACC patients (R1 or Rx resection or in stage III) was suggested consideration of RT according to the current recommendations (24). Registry analyses using National Cancer Database (NCD) and the National Cancer Institute’s Surveillance, Epidemiology, and End Results (SEER) database also have resulted in conflicting conclusions to the benefit of adjuvant radiation (25, 26). A recent population-based publication on the role of adjuvant RT for nonmetastatic ACC used the SEER Database and included 365 patients (27). Only 55(15.1%) of patients received adjuvant RT, patients who had adjuvant RT were more likely to be higher-disease stage patients. Adjuvant radiation improved overall survival with a 48% decreased risk of death for all patients and may confer a survival benefit only in patients with a high risk of recurrence. However, these studies are limited by the lack of resection status, heterogeneous treatment quality. Single-institution analyses provides a source of homogeneous patient populations for unified treatment through rigorous treatment evaluations. In our study, ACC patients are treated with guidance from multidisciplinary team with expertise in endocrine malignancies. The multivariate analysis indicates that, after adjusting for Ki67 and stage, adjuvant RT did not demonstrate to be independently associated with improved DFS. However, in the subgroup analysis for tumor characteristics (Ki67), adjuvant RT didn’t improve the survival outcomes compared with nonmetastatic ACC who only underwent radical surgery (**Supplementary Figure 1**). The majority of the participants were ENSAT II, we found that they can also benefit from adjuvant RT. Although we have found that ACC patients who received adjuvant RT had better survival outcomes in definitive ENSAT II, ENSAT I/II and ENSAT I/II/III subgroup, we were unable to independently analyze ENSAT I and ENSAT III data, due to the limited sample size.

Side effects of RT mainly include fatigue, nausea, diarrhea, potential kidney/liver damage, and obstruction (28). Besides, the side effects of mitotane include nausea, fatigue, anorexia, and diarrhea (29). Given the overlap of these side effects, patients may experience nausea and diarrhea while undergoing RT. Therefore, the true rate of side effects might be even lower. Our results suggested ACC patients tend to tolerate RT well.

There are several limitations of this retrospective study. There are still a limited number of study subjects. The analysis was restricted to the collected variables and the accuracy of the recorded data. Therefore, some factors that may contribute to patient outcomes are not available. We believe patients with postoperative adjuvant RT had improved 3-year OS and DFS compared with surgery alone in resected ACC patients, especially for patients with ENSAT I/II stage. Although this retrospective study on RT in localized ACC provides the evidence that RT has certain value in ACC, its findings need to be prospectively confirmed.

## Data availability statement

The original contributions presented in the study are included in the article/**Supplementary Material**. Further inquiries can be directed to the corresponding author.

## Ethics statement

The studies involving humans were approved by Ruijin Hospital, Shanghai Jiao Tong University School of Medicine. The studies were conducted in accordance with the local legislation and institutional requirements. The participants provided their written informed consent to participate in this study.

## Author contributions

LW: Data curation, Funding acquisition, Writing – original draft, Formal analysis, Investigation, Methodology, Project administration. JC: Data curation, Methodology, Project administration, Writing – original draft. TS: Investigation, Writing – review & editing. LJ: Data curation, Investigation, Writing – review & editing. YH: Conceptualization, Methodology, Writing – review & editing. CZ: Conceptualization, Data curation, Formal analysis, Investigation, Writing – original draft. WZ: Data curation, Writing – original draft. YJ: Investigation, Software, Writing – original draft. XZ: Validation, Writing – original draft. WW: Funding acquisition, Project administration, Writing – review & editing.

## References

[1] Crona J Beuschlein F. Adrenocortical carcinoma-towards genomics guided clinical care. Nat Rev Endocrinol (2019) 15:548–60. doi: 10.1038/s41574-019-0221-7 31147626

[2] Miller Barbra S Doherty Gerard M. Surgical management of adrenocortical tumours. Nat Rev Endocrinol (2014) 10:282–92. doi: 10.1038/nrendo.2014.26 24637859

[3] Berruti A Fassnacht M Haak H Else T Baudin E Sperone P. Prognostic role of overt hypercortisolism in completely operated patients with adrenocortical cancer. Eur Urol (2014) 65:832–8. doi: 10.1016/j.eururo.2013.11.006 24268504

[4] Libé R Borget I Ronchi CL Zaggia B Kroiss M Kerkhofs T. Prognostic factors in stage III-IV adrenocortical carcinomas (ACC): an European Network for the Study of Adrenal Tumor (ENSAT) study. Ann Oncol (2015) 26:2119–25. doi: 10.1093/annonc/mdv329 26392430

[5] Wu L Xie J Jiang L Su T Ye L Zhou W. Feminizing adrenocortical carcinoma: the source of estrogen production and the role of adrenal-gonadal dedifferentiation. J Clin Endocrinol Metab (2018) 103:3706–13. doi: 10.1210/jc.2018-00689 30053001

[6] Fassnacht M Assie G Baudin E Eisenhofer G de la Fouchardiere C Haak HR. Adrenocortical carcinomas and Malignant phaeochromocytomas: ESMO-EURACAN Clinical Practice Guidelines for diagnosis, treatment and follow-up. Ann Oncol (2020) 31:1476–90. doi: 10.1016/j.annonc.2020.08.2099 32861807

[7] Calabrese A Puglisi S Borin C Basile V Perotti P Pia A. The management of postoperative disease recurrence in patients with adrenocortical carcinoma: a retrospective study in 106 patients. Eur J Endocrinol (2023) 188:118–24. doi: 10.1093/ejendo/lvad002 36655273

[8] Habra MA Ejaz S Feng L Das P Deniz F Grubbs EG. A retrospective cohort analysis of the efficacy of adjuvant radiotherapy after primary surgical resection in patients with adrenocortical carcinoma. J Clin Endocrinol Metab (2013) 98:192–7. doi: 10.1210/jc.2012-2367 PMC3537094 23150683

[9] Kasperlik-Załuska AA Migdalska BM Zgliczyński S Makowska AM. Adrenocortical carcinoma. A clinical study and treatment results of 52 patients. Cancer (1995) 75:2587–91. doi: 10.1002/1097-0142(19950515)75:10<2587::AID-CNCR2820751028>3.0.CO;2-5 7736405

[10] Bodie B Novick AC Pontes JE Straffon RA Montie JE Babiak T. The Cleveland Clinic experience with adrenal cortical carcinoma. J Urol (1989) 141:257–60. doi: 10.1016/S0022-5347(17)40734-8 2913342

[11] Viani GA Viana BS. Adjuvant radiotherapy after surgical resection for adrenocortical carcinoma: A systematic review of observational studies and meta-analysis. J Cancer Res Ther (2019) 15:S20–6. doi: 10.4103/jcrt.JCRT_996_15 30900615

[12] Bedrose S Daher M Altameemi L Habra MA. Adjuvant therapy in adrenocortical carcinoma: reflections and future directions. Cancers (Basel) (2020) 12:508. doi: 10.3390/cancers12020508 32098326 PMC7072549

[13] Polat B Fassnacht M Pfreundner L Habra MA. Radiotherapy in adrenocortical carcinoma. Cancer (2009) 115:2816–23. doi: 10.1002/cncr.24331 19402169

[14] Nelson DW Chang S-C Bandera BC Fischer Trevan D Wollman R Goldfarb M. Adjuvant radiation is associated with improved survival for select patients with non-metastatic adrenocortical carcinoma. Ann Surg Oncol (2018) 25(7):2060–6. doi: 10.1245/s10434-018-6510-x 29748889

[15] Else T Williams AR Sabolch A Jolly S Miller BS Hammer GD. Adjuvant therapies and patient and tumor characteristics associated with survival of adult patients with adrenocortical carcinoma. J Clin Endocrinol Metab (2014) 99:455–61. doi: 10.1210/jc.2013-2856 PMC3913818 24302750

[16] Kimpel O Schindler P Schmidt-Pennington L Altieri B Megerle F Haak H. Efficacy and safety of radiation therapy in advanced adrenocortical carcinoma. Br J Cancer (2023) 128:586–93. doi: 10.1038/s41416-022-02082-0 PMC9938283 36482186

[17] Fassnacht M Hahner S Polat B Koschker A-C Kenn W Flentje M. Efficacy of adjuvant radiotherapy of the tumor bed on local recurrence of adrenocortical carcinoma. J Clin Endocrinol Metab (2006) 91:4501–4. doi: 10.1210/jc.2006-1007 16895957

[18] Ginsburg KB Chandra AA Schober JP Handorf EA Uzzo RG Greenberg RE. Identification of oncological characteristics associated with improved overall survival in patients with adrenocortical carcinoma treated with adjuvant radiation therapy: Insights from the National Cancer Database. Urol Oncol (2021) 39:791.e1–e7. doi: 10.1016/j.urolonc.2021.06.019 34301459

[19] Zhu J Zheng Z Shen J Lian X Miao Z Shen J. Efficacy of adjuvant radiotherapy for treatment of adrenocortical carcinoma: a retrospective study and an updated meta-analysis. Radiat Oncol (2020) 15:118. doi: 10.1186/s13014-020-01533-3 32448148 PMC7245885

[20] Abdel-Rahman O. Impact of postoperative radiotherapy on the outcomes of resected adrenocortical carcinoma-a real-world, population-based study. Strahlenther Onkol (2022) 198(1):73–9. doi: 10.1007/s00066-021-01838-6 34476529

[21] Fassnacht M Johanssen S Fenske W Weismann D Agha A Beuschlein F. Improved survival in patients with stage II adrenocortical carcinoma followed up prospectively by specialized centers. J Clin Endocrinol Metab (2010) 95:4925–32. doi: 10.1210/jc.2010-0803 20668036

[22] Sabolch A Else T Griffith KA Ben-Josef E Williams A Miller BS. Adjuvant radiation therapy improves local control after surgical resection in patients with localized adrenocortical carcinoma. Int J Radiat Oncol Biol Phys (2015) 92:252–9. doi: 10.1016/j.ijrobp.2015.01.007 25754631

[23] Gharzai LA Green MD Griffith KA Else T Mayo CS Hesseltine E. Adjuvant radiation improves recurrence-free survival and overall survival in adrenocortical carcinoma. J Clin Endocrinol Metab (2019) 104:3743–50. doi: 10.1210/jc.2019-00029 PMC8926022 31220287

[24] Fassnacht M Dekkers O Else T Baudin E Berruti A de Krijger R. European Society of Endocrinology Clinical Practice Guidelines on the management of adrenocortical carcinoma in adults, in collaboration with the European Network for the Study of Adrenal Tumors. Eur J Endocrinol (2018) 179:G1–G46. doi: 10.1530/EJE-18-0608 30299884

[25] Li Y Bian X Ouyang J Wei S He M Luo Z. Nomograms to predict overall survival and cancer-specific survival in patients with adrenocortical carcinoma. Cancer Manag Res (2018) 10:6949–59. doi: 10.2147/CMAR.S187169 PMC6300377 30588100

[26] Thomas Justin J Tward Jonathan D. Stage presentation. Care patterns, treatment outcomes, and impact of radiotherapy on overall survival for adrenocortical carcinoma. Clin Genitourin Cancer (2021) 19:417–24. doi: 10.1016/j.clgc.2021.03.009 33858789

[27] Wu K Liu X Liu Z Lu Y Wang X Li X. Benefit of postoperative radiotherapy for patients with nonmetastatic adrenocortical carcinoma: A population-based analysis. J Natl Compr Canc Netw (2021) 19:1425–32. doi: 10.6004/jnccn.2021.7035 34902831

[28] Franzese C Stefanini S Massaro M Comito T Navarria P Clerici E. Phase II trial of stereotactic body radiation therapy on adrenal gland metastases: evaluation of efficacy and impact on hormonal production. J Cancer Res Clin Oncol (2021) 147:3619–25. doi: 10.1007/s00432-021-03807-z PMC11802008 34537907

[29] Veytsman I Nieman L Fojo T. Management of endocrine manifestations and the use of mitotane as a chemotherapeutic agent for adrenocortical carcinoma. J Clin Oncol (2009) 27:4619–29. doi: 10.1200/JCO.2008.17.2775 PMC2754909 19667279

