# Supplementary figures and images for: Efficacy and safety of adjuvant radiation therapy in localized adrenocortical carcinoma
Source: Front Endocrinol (Lausanne). 2024 Jan 8;14:1308231. doi: 10.3389/fendo.2023.1308231 (PMC10801189; doi:10.3389/fendo.2023.1308231)

**Supplementary Figure 1. Effect of adjuvant RT on survival outcomes and DFS in patients with Ki67(>10%).**

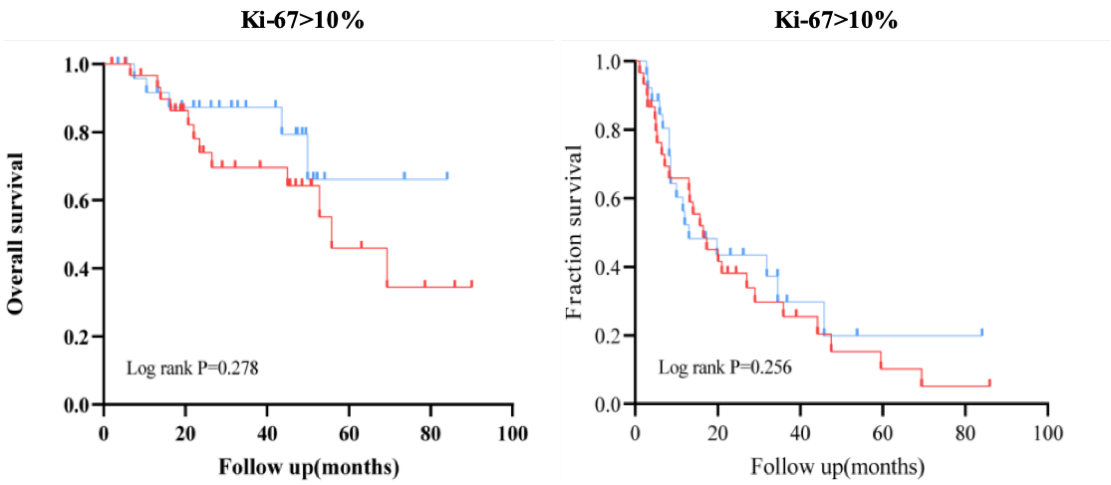

Supplement: Supplementary file 1 [file Image_1.pdf]
